# Extracting regulatory modules from gene expression data by sequential pattern mining

**DOI:** 10.1186/1471-2164-12-S3-S5

**Published:** 2011-11-30

**Authors:** Mingoo Kim, Hyunjung Shin, Tae Su Chung, Je-Gun Joung, Ju Han Kim

**Affiliations:** 1 Seoul National University Biomedical Informatics (SNUBI), Seoul National University College of Medicine, Seoul 110799, Korea; 2 Dept. of Industrial and Information Systems Engineering, Ajou University, Suwon 443749, Korea; 3 Systems Biomedical Informatics National Core Research Center, Seoul National University College of Medicine, Seoul 110799, Korea; 4 Institute of Endemic Diseases, Seoul National University College of Medicine, Seoul 110799, Korea; 5 Div. of Biomedical Informatics, Seoul National University College of Medicine, Seoul 110799, Korea

## Abstract

**Background:**

Identifying a regulatory module (RM), a bi-set of co-regulated genes and co-regulating conditions (or samples), has been an important challenge in functional genomics and bioinformatics. Given a microarray gene-expression matrix, biclustering has been the most common method for extracting RMs. Among biclustering methods, order-preserving biclustering by a sequential pattern mining technique has native advantage over the conventional biclustering approaches since it preserves the order of genes (or conditions) according to the magnitude of the expression value. However, previous sequential pattern mining-based biclustering has several weak points in that they can easily be computationally intractable in the real-size of microarray data and sensitive to inherent noise in the expression value.

**Results:**

In this paper, we propose a novel sequential pattern mining algorithm that is scalable in the size of microarray data and robust with respect to noise. When applied to the microarray data of yeast, the proposed algorithm successfully found long order-preserving patterns, which are biologically significant but cannot be found in randomly shuffled data. The resulting patterns are well enriched to known annotations and are consistent with known biological knowledge. Furthermore, RMs as well as inter-module relations were inferred from the biologically significant patterns.

**Conclusions:**

Our approach for identifying RMs could be valuable for systematically revealing the mechanism of gene regulation at a genome-wide level.

## Background

Identifying regulatory modules (RMs) and their interaction networks has been an important challenge in functional genomics and bioinformatics [1-4]. Given a microarray gene-expression matrix, comprised of the rows of genes and the columns of samples (or conditions), biclustering has been the most common method extracting RMs defined as a bi-set of co-regulated genes and coregulating conditions [5-11]. Biclustering simultaneously performs row–clustering and column–clustering, according to the similarities among expression profile vectors of genes and of samples, respectively.

Biclustering has two favorable properties to conventional clustering. First, a gene can be assigned to multiple clusters. This property is well fit to complex biological processes, i.e. a gene can participate in more than one biological process. Second, only the selected conditions are considered for clustering. This property is also in suit with biological implication since genes are only co-regulated under specific conditions. There have been various approaches for biclustering according to the variety of homogeneity definition and search strategy [12]. Cheng and Church [6] define a bicluster as a submatrix having a low mean squared residue score. A bicluster is found from a random seed per iteration and the entries of the cluster are replaced with a random number. Since the biclusters are identified separately in a random fashion, the result may represent an arbitrary subset of all biclusters depending on iterations. Furthermore, the random replacement may interfere with the subsequent identification of biclusters.

FLOC (Flexible Overlapping biClutering) algorithm improves Cheng and Church (2000)’s algorithm by avoiding the random interference and simultaneously discovering a set of *k-*possibly–overlapping–biclusters [13]. The number of biclusters, *k*, however, is arbitrarily specified without considering the inherent characteristics of input data. Ben-Dor et al. [5] focus on the relative order of gene expression values. They define an order-preserving bicluster as a group of genes whose values induce same linear ordering across the samples in a sample subset. They proposed a search algorithm (Order Preserving SubMatrix, OPSM) based on a probabilistic model for a single order-preserving submatrix. The OPSM method, however, is also limited to find each bicluster separately and does not show enough sensitivity to find hidden biclusters.

Recently, there has been an approach to identify order-preserving biclusters using sequential pattern mining (SPM), which has been intensively studied in the field of data mining [14]. SPM is to discover frequent sub-sequences as patterns in a sequence database. In search for order-preservation, it provides an advantage of sensitivity through exhaustive enumeration of all possible patterns. However, SPM is highly time– and memory–demanding especially under gene expression data. Its complexity grows exponentially with the length of sequence whereas linearly with the number of sequences [15]. Note that a typical microarray data has a large gene–wise dimension and can have a large sample–wise one in case of combined datasets, Therefore, it can easily be computationally intractable to apply the standard SPM algorithms to the huge-sized gene expression data in practice. Another difficulty with SPM is related to inherent noise in microarray data. SPM attempts to find an exact order of genes (or conditions) according to the magnitude of the expression value. However, there almost always exists experimental noise in microarray data, which may cause trivial changes in ordering the expression values. In such a case, the current version of SPM can easily fail to detect significant biological patterns since some of the genes are not in due exact order.

In this paper, we propose a novel sequential pattern mining algorithm. This algorithm has several attractive features comparing with the related work: it is scalable in the size of microarray data and robust with respect to noise. When applied to the microarray data of yeast, the proposed algorithm successfully found long order-preserving patterns, which are biologically significant but cannot be found in randomly shuffled data. The resulting patterns are well enriched to known annotations (about genes and conditions as well) and are consistent with known biological knowledge. Among the patterns, the biologically significant patterns were used to infer RMs. There can be more interesting relationships on the level of module, and so the inter-relations between the resulting RMs were further examined. They were categorized into one of four types including (1) independent; (2) conditionally co-regulated; (3) separately co-regulated; and (4) similar. The respective types of inter-module relations were exemplified with biological inferences via enrichment study.

## Results

### Comparison with other methods in sequential pattern mining

**Figure 1:**
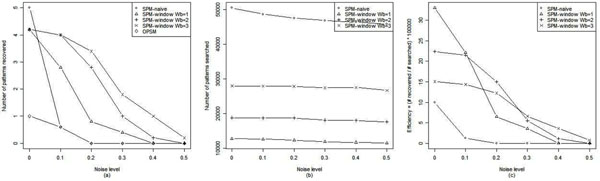
**Benchmark plots.** Benchmark plots: comparison of (a) sensitivity (b) search complexity and (c) efficiency in various noise levels. Values are averaged over five replications. Five order-preserving patterns of ℜ^|10|×|10|^ are embedded to a random matrix of ℜ^|50|×|50|^ . The search parameters are set to ***u*** = 10, ***l*** = 10 for both SPM-window and SPM-naive, and ***w_f_*** = 20, ***w_b_*** = 1, 2, 3 for SPM-window.

The proposed sequential pattern mining, sequential pattern mining with search windows (SPM-window) is compared to previous techniques. Candidates for comparison are the OPSM method of Ben-Dor *et al.*, 2003 and the naïve application of sequential pattern mining without search windows (SPM-naive) [5,14]. Other biclustering methods are not considered since they are not targeting order-preserving patterns. For performance comparison, the algorithms are tested on simulation data with embedded sequential patterns. SPM-based algorithms perform better than OPSM in terms of the sensitivity to hidden patterns (Fig.1a). Basically, SPM-naive misses no patterns since it performs an exhaustive enumeration of all possible patterns. The perfect sensitivity of SPM, however, comes with the cost of complexity. The time complexity of SPM is more than exponential and easily becomes intractable as data get larger. Lowering the time complexity is inevitable when one wants to search real-sized gene expression data. As can be seen in Fig.1b and Fig.1c, SPM-window is more efficient than SPM-naive finding as many patterns as its naive counterpart while searching much less. For the reality of microarray condition, the algorithms are tested with addition of noise to simulation data. The sensitivity of SPM-naïve decreases quickly as the noise increases while SPM-window shows better sensitivity and efficiency within a reasonable range of noise (Fig.1). We used BicAT for the implementation of OPSM [16]. Further details of benchmark, such as the generation of simulation data and the parameter selection, are shown in Additional File 1.

### Searching of sequential patterns

We applied the proposed sequential pattern mining algorithm to an expression matrix ***E*** consisting 3,860 genes and 1,050 samples by combining all available yeast cDNA datasets from the Stanford Microarray Database (SMD) [17]. We found 3,250,198 sequential patterns, |***P***| = 3, 250, 198. The ***P*** comprises 538 unique genes out of 3,860 and 1,009 unique samples out of 1,050. Table 1 shows the distribution of the sequential patterns with respect to the length ***δ^g^*** and the support ***δ***^s^. The parameters (see Methods section) were set ***u*** = 32, ***l*** = 6, ***w_f_*** = 535, and ***w_b_*** = 53. The number in each cell indicates the frequency for the corresponding combination of (***δ^g^***, ***δ***^s^) where ***δ^g^*** = 6, . . . , 9 and ***δ****^s^* = 32, . . . , 53. The minimum support threshold ***u*** was set to 3% of the total number of samples, and the size of backward lookup ***w_b_*** was set to 10% of ***w_f_*** . The minimum length threshold l and the size of forward lookup ***w_f_*** were empirically set. To validate the significance of resulting sequential patterns, we re-ran the same algorithm to the same data but the data elements were re-shuffled. As a result, no pattern was found all through the ten times of experimental replications. This indicates the sequential patterns only exist in biological data, which justifies the biological significance of our results.

**Table 1:** Distribution of sequential patterns

length \ support	6	7	8	9	Total
32	1,107,247	98,991	1,359	3	1,207,600
33	711,700	49,900	351		761,951
34	454,088	24,350	73		478,511
35	290,648	11,614	14		302,276
36	184,029	5,270			189,299
37	117,035	2,402			119,437
38	72,871	926			73,797
39	45,702	403			46,105
40	28,397	138			28,535
41	17,560	44			17,604
42	10,815	5			10,820
43	6,234	4			6,238
44	3,657				3,657
45	2,055				2,055
46	1,154				1,154
47	572				572
48	286				286
49	164				164
50	79				79
51	38				38
52	13				13
53	7				7

Total	3,054,351	194,047	1,797	3	3,250,198

Distribution of sequential patterns with respect to length ***δ^g^*** and the support ***δ***^s^

### Functional validation of sequential patterns

#### Significance test by enrichment study

**Figure 2:**
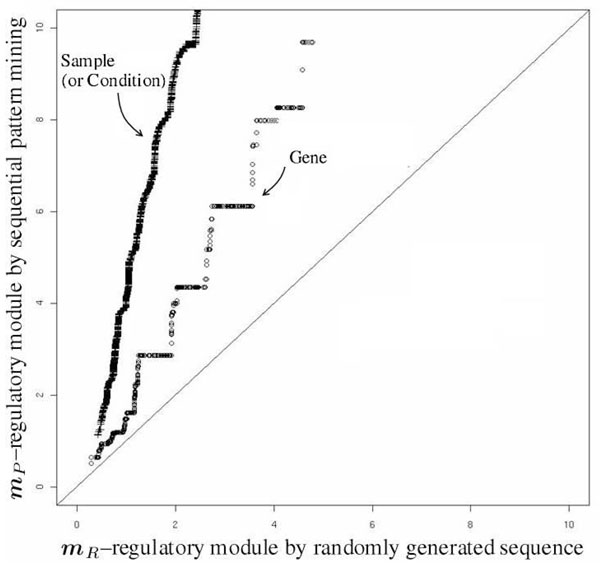
**Quantile-quantile plot**. Quantile-quantile plot: The pairs of p-values corresponding to the pairs of (***m_P_***, ***m_R_***) on the same quantile are scattered onto the axes of negative log-scale. The circle stands for the p–values of gene annotation, whereas the cross stands for the p–values of sample annotation.

RMs, co-regulated and their co-regulating samples were identified from the sequential patterns. Biological relevance of the RMs was evaluated by enrichment study. First, a set ***R*** of random sequential patterns was generated as a competitor against ***P***. For each cell of (***δ^g^***, ***δ^s^***) in Table 1, the ***δ^g^*** – sequences of randomly permuted gene-names were generated as the same quantity of (***δ^g^***, ***δ***^s^). Second, all the RMs, both ***m_P_*** ’s from ***P*** and ***m_R_***’s from ***R***, were enriched to known annotations by calculating hypergeometric distributions in regard to GO-slim terms and SMD-sample categories, respectively. The genes were annotated by 33 Gene Ontology (GO) slim terms (of Biological Process) defined by SGD. And, the samples were annotated by 33 SMD categories which describe the experimental context of microarray samples. More details of GO-slim terms and SMD-sample categories are shown in Additional File 2. Then, among the terms (or categories) corresponding to the genes (or the samples) of a RM, the p–value of the most significant term (category) was allocated to the regulatory module [9]. Third, the p-values of ***m_P_*** and ***m_R_*** were compared. Fig. 2 presents the quantile-quantile plot of the p–value distributions of both ***m_P_*** and ***m_R_***. The large departure toward vertical axis from the diagonal line indicates ***m_P_*** is more significantly enriched than the competitor ***m_R_***. The circle stands for the p–value of gene annotation, whereas the cross stands for the p–value of sample annotation.

#### Biological implication of regulatory modules

From 3,250,198 RMs, 426,558 modules were selected, each of which has a p-value less than 0.001 with respect to the most significant annotation (The expected false discovery rate of the threshold (p = 0.001) is about 8%). Only 26,444 ***m_R_***’s were significant in the randomly generated modules whereas 426,558 ***m_P_*** ’s in the modules by sequential pattern mining. Table 2 shows biological implication of the selected RMs by relating the gene context (in terms of GO-slim term) and the sample context (in terms of SMD category). Each cell contains the number of the RMs belonging to the specified context by both aspects. For simplicity, trivial GO-slim terms (or SMD categories) having no *RM* were removed from the table. Note the two prominent couplings: “[GO] generation of precursor metabolites and energy– [SMD] RNA processing,” and “[GO] cell homeostasis – [SMD] oxidative stress,” which imply the existence of co-regulation under a specific biological condition.

**Table 2:** Biological implication of regulatory modules

GO-slim	SMD category	Calcium	Salt treatment	Mutants	Oxidative stress	RNA processing	Stress	DNA damage	Nutrient effects	Chemical effects	Chemostat	Starvation	Mutant vs Wild-type	Total
Response to stress		154,651	142,140	28,467	16,889	23	3,230	527	462	29	2	18	0	346,438
Carbohydrate metabolism		15,360	19,295	19,747	1,534	0	1,545	156	6	196	18	0	5	57,862
Generation of precursor metabolites and energy		222	1,186	399	37	**15,692**	149	0	0	0	0	0	0	17,685
Biological process unknown		2,959	911	129	0	0	25	0	0	0	0	0	0	4,024
Cell homeostasis		0	0	0	**514**	0	0	0	0	0	0	0	0	514
Cellular respiration		0	0	1	13	0	0	0	0	0	0	0	0	14
Electron transport		6	0	0	0	0	1	0	0	0	0	0	0	7
Lipid metabolism		0	1	0	5	0	0	0	0	0	0	0	0	6
Amino acid and derevative metabolism		0	6	0	0	0	0	0	0	0	0	0	0	6
Sporulation		2	0	0	0	0	0	0	0	0	0	0	0	2

Total		173,200	163,539	48,743	18,992	15,715	4,950	683	468	225	20	18	5	426,558

Regulatory modules: the cell contains the number of the RMs belonging to the specified context by both GO-slim term and SMD category

### Identification of inter-module relations

#### Distribution of module overlap

**Figure 3:**
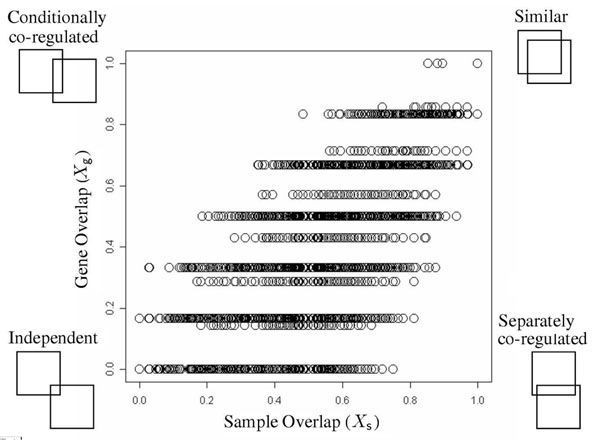
**Inter-modular relation by gene- vs. sample- overlap.** Inter-modular relation by gene- vs. sample- overlap: All possible pairs of 300 representative *RMs*, _300_*C*_2_ pairs, are scattered onto the panel of ***X_g_***–axis and ***X_s_***–axis. A circle represents (***X_s_***, ***X_g_***) of a pair (***m***, ***m′***). The cartoons at the corners represent the four types of inter-module relations; the disposition of two boxes illustrates how two modules are relatively located in the gene expression matrix, where a box symbolizes a module ***m*** with the rows of genes and the columns of samples.

Given different RMs, ***m*** and ***m′***, we can infer the inter-module relation between them based on the two dimensional degrees of overlap, ***X_g_*** and ***X_s_***.(see Methods section). For the sake of representational convenience, we sampled 300 representatives out of the original 426,558 RMs; 300 centroids from k-medoid clustering (k = 300) were selected as the representative modules. The k-medoid clustering is based on the distance between RMs, defined as an inverse of the proportion of overlap area to the area sum of two RMs. Among the representative modules, 249 were most significantly enriched to ‘response to stress’ of GO-slim term, 34 to ‘carbohydrate metabolism’, 12 to ‘generation of precursor metabolites’ and energy’, 4 to ‘biological process unknown’, and 1 to ‘cell homeostasis’. Fig. 3 shows the plot of (***X_s_*, *X_g_***)’s for all possible pairs of (***m***,***m′***). Most of the pairs belong to either similar or independent type since the gene sets of two modules are similar (dissimilar), and the sample set are highly likely to be similar (dissimilar) as well. However, in the viewpoint of biology, it will be more interesting to investigate the remaining two types of inter-module relations, conditionally co-regulated and separately co-regulated. The relationship of conditionally co-regulated may be observed when a gene set is controlled by different regulation mechanisms, and maybe at different biological states. On the other hand, the relationship of separately co-regulated may happen if distinct gene sets are co-regulated separately under same samples.

#### Typical examples of four inter-module relations

**Figure 4:**
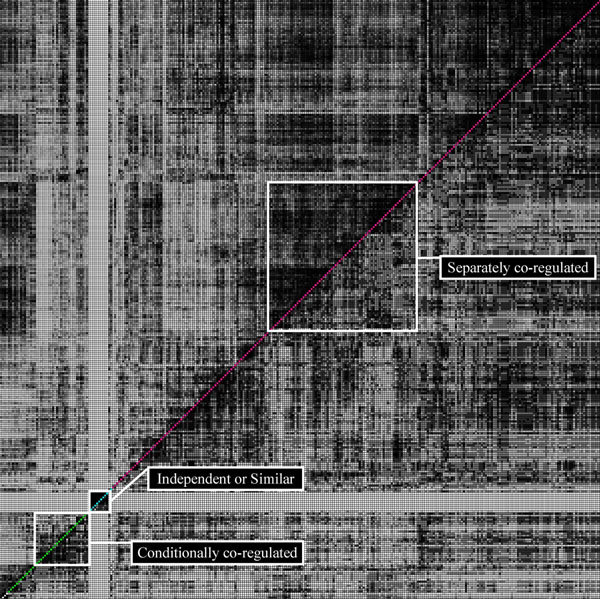
**Inter-module relations of 300 modules.** Inter-module relations of 300 modules: the coordinate (*i*, *j*) in lower diagonal where *i* >*j* (1 ≤ *i*, *j* ≤ 300) represents the gene overlap (***X_g_***) between ***m_i_*** and ***m_j_*** . On the other hand, the upper diagonal coordinate where *i* <*j* represents the sample overlap (***X_s_***). The gray-scale indicates the degree of overlap; the darker the higher overlap. Each square groups the modules which belong to the same type of inter-module relations; independent, conditionally co-regulated, separately co-regulated, and similar. The modules in the square tagged independent or similar are characterized as ‘externally exclusive’ to other (outside) modules but ‘internally similar’ with the modules grouped together. Both aspects of independent and similar are represented as darker gray-values within the square but lighter ones outside the square. It implies the genes are tightly associated only with specific samples (experimental conditions).

Fig. 4 visualizes the inter-module relations of the representative modules. For the ease of representation, the modules were rearranged in order that similar samples within an identical GO-slim term were grouped together. In the plot, the coordinate (*i*, *j*) in the lower diagonal where *i* >*j* (1 ≤ *i*, *j* ≤ 300) represents the gene overlap (***X_g_***) between ***m_i_*** and ***m_j_*** , on the other hand, the coordinate (*i*, *j*) in the upper diagonal where *i* <*j* represents the sample overlap (***X_s_***). The gray-scale indicates the degree of overlap; the darker the higher overlap. The square roughly groups the modules which belong to the same type of inter-module relation. The following describes group-wise characteristics of the modules.

**Type 1 / type 4 (independent/similar)**: The modules in the square tagged independent or similar correspond to Type 1 or Type 4, respectively. A module in the square has independent relationship with external modules–the modules outside the square. Both the gene set and the sample set of the module are exclusive from those of external modules, leading to small values of ***X_g_*** and ***X_s_***. On the contrary, modules within the square are similar with other internal modules. They highly share the gene set and the sample set, thus the values of ***X_g_*** and ***X_s_*** are large. Both aspects of independent and similar are represented as darker gray-values within the square but lighter ones outside the square. They share the genes, YGR254W, YGR192C, YKL060C, and YJR009C (more than 7 out of 9 modules), which all participate in ‘glucose metabolism’. These genes are not included in the external modules. The sample context, on the other hand, is characterized by the ‘RNA processing’; 96% of the samples in internal modules are related to ‘RNA processing’ while only 0.08% are in external modules. Therefore, it is suggested that the four genes associated with ‘glucose metabolism’ are bound by ‘RNA processing’.

**Type 2 (conditionally co–regulated)**: The modules in the square tagged conditionally co–regulated belong to Type 2. The darker gray-values of the lower diagonal matrix contrast to the lighter ones of the upper diagonal matrix. This implies that they are highly overlapped in genes but little in samples. More than half of the modules include YMR105C, YBR126C, and YFR053C, which are all related to ‘carbohydrate metabolism’ as ‘Phosphoglucomutase’, ‘Trehalose-6-Phosphate synthase’, and ‘Hexokinase I’, respectively. However, the sample contexts are as diverse as ‘oxidative stress’,‘DNA damage’, ‘Nutrient effects’, ‘Chemical effect’, and so on. Therefore, it is suggested that those three genes related to ‘carbohydrate metabolism’ are conditionally co-regulated depending on different conditions.

**Type 3 (separately co–regulated)**: The modules in the square tagged separately co–regulated belong to Type 3. Opposite to Type 2, the square is light gray-valued in the lower diagonal contrasting to the dark ones in the upper diagonal. This represents that the modules are highly overlapped in samples but little in genes. The samples are dominated by ‘stress or calcium’. However, the representative context of genes is hardly identified.

## Discussion

In this paper, we successfully demonstrated the existence of sequential patterns in gene expression data and validated their biological significance. Furthermore, we inferred inter-module relations based on module overlap and illustrated the examples of condition-specific co-regulations. Module groups in overlap plot also suggest the hierarchical organization of module where genes are grouped into modules that are clustered into supermodules [18]. Our approach could be improved for identifying modular structure in biological system, regarding following several points.

First, we can score the strength of gene order instead of binary decision of their order. This scoring can be achieved by testing the hypothesis that the differential expression of gene A is same to the differential expression of gene B. Second, samples can have different weights. Each biological condition set has different sample resolution: some conditions are studied with large number of microarrays while others are rarely observed due to several reasons including technical difficulty. Although it is difficult to get equal resolution as we integrate more datasets, alternative approaches may be applied. For example, with a weighting scheme of samples - higher weight for rare samples and lower otherwise, patterns can be represented in more diverse biological conditions.

Although our approach has several advantages it still needs more improvements. The first one is the scalability of algorithm. We used yeast expression data of which the size is not relatively large in order to demonstrate the efficiency of our algorithm. In further study, we will try to deal with the pattern mining problem using huge microarray dataset consisting of human and mouse gene expression profiles in diverse conditions. To solve this problem, more memory-efficient algorithm is needed. The second is about running speed. In this study, we mainly focused on the ability of finding modules without considering the running time. However, the running time can be significantly decreased using efficient data structure such as suffix tree.

## Conclusions

In this paper, we presented a sequential pattern mining algorithm to identify RMs from microarray data. The existing definition for sequential pattern was relaxed so that the algorithm can fit into the biological implication in hand. The searching method was also modified to be more scalable and flexible than previous one. The biological meanings of the resulting RMs were enriched through known annotations for genes and samples as well. In addition, the types of relations between modules were further investigated; based on the degree of overlap between two modules, the relation was categorized into one of the four types: 1) independent, 2) conditionally co-regulated, 3) separately co-regulated, and 4) similar. Our approach enables a systematic study of inter-module relations beyond the identification of single module in gene regulation. The research on modular relations in gene expression will be valuable for revealing the mechanism of gene regulation.

## Methods

### Experimental dataset

An expression matrix ***E*** was created with 3,860 genes and 1,050 samples by combining all available yeast cDNA datasets from the Stanford Microarray Database (SMD) [17]. We used 3,860 out of the 4,356 genes verified in Saccharomyces Genome Database (SGD) [19]. We excluded ribosome-related genes because they have exceptionally high tendency for order-preservation in a preliminary analysis. Specifically, we excluded those genes annotated to two specific Gene Ontology terms: GO:0042254 (ribosome biogenensis and assembly) in the Biological Process branch and GO:005840 (ribosome) in the Cellular Component branch. Their exceptional richness in order-preserving tendency, which may originate from the fact that they comprise huge complexes, overwhelms that of those in other categories. Removing ribosomal genes effectively reveals weaker signals from other genes and greatly reduces search complexity.

### Sequential pattern mining with gene order preservation

A gene expression matrix ***E*** is a matrix of the absolute value of log-ratio of expression level of gene *i* under a specific sample *j*. A zero value of ***E****_ij_* indicates no differential expression of gene *i* in sample *j*, and ***E****_ij_* >***E****_kj_* indicates that gene *i* is more differentially expressed than gene *k* in sample *j*. The matrix ***E*** is then transformed to a sequence database, ***D***. The genes of sample *j* in ***D*** are sorted by descending order of expression value of ***E****_ij_*, resulting a sequence of genes per sample. In this section, we introduce the modified sequential pattern mining algorithm based on the sequence.

The notations used for definitions are as following:

***G*** A set of genes, ***G*** = {*g*_1_, *g*_2_, . . . , *g*_|_*_G_*_|_}, *i* = 1, . . . , |***G***|.

***S*** A set of samples, ***S*** = {*s*_1_, *s*_2_, . . . , *s*_|_*_S_*_|_}, *j* = 1, . . . , |***S***|.

***E*** An expression matrix, ***E*** ∈ ℜ^|^***^G^***^|×|^***^S^***^|^.

***D*** A sequence database from ***E***. Each sequence is a gene-sequence of length |***G***|, ***D*** = {***d***_1_, ***d***_2_ ,. . . , ***d***_|_***_S_***_|_}

***d****_j_* A sequence of genes in sample *j*. The length of ***d****_j_* is |***G***|

***p*** A sequential pattern.

***p***_g_ A sequence of genes in ***p***. The length of ***p*** is |***p***_g_|.

***p***_s_ A subset of samples in ***p***. The support of ***p*** is |***p***_s_|.

***l*** The user-specified threshold for the minimum length.

***u*** The user-specified threshold for the minimum support.

Our goal is to find a sequential pattern with the longest length of gene sequence and the largest samples size simultaneously, supporting a common set of samples. Here we propose a novel sequential pattern mining algorithm that finds long order preserving patterns.

**Definition 1.** Let *i* be a reference point of the given sequence of length *n*,

***d*** = *g*^1^*g*^2^ … *g^i^* … *g^n^*^−1^*g^n^*

A generalized sequence of length 2, *g^i^g^k^* is constructed if *k* satisfies

*k* ∈ [ *i*−***w_b_****i*+***w_f_*** ], *k* ≠ *i*, 1 ≤ *i* ≤ *n*−1,

where ***w_b_*** and ***w_f_*** specify the sizes of backward- and forward- windows (0 ≤ ***w_b_*** ≤ *i*−1, 1≤ ***w_f_*** ≤ *n*−*i*), respectively. The size of sequence searching window ***w*** is therefore ***w_f_*** +***w_b_***. A longer (generalized) sequence of length |***p***_g_| (> 2) is similarly expanded by moving the reference point *i* to *k* (*i* ← *k* if *k* >*i*, *i* ← *i* otherwise) and padding a new *g^k^* up to length |***p***_g_|. If a generalized sequence arises more than *u* times in ***D***, i.e., |***p***_s_| ≥ *u*, with more forward occurrences than backward ones, we define it as a **sequential pattern *p***. The definition of sequential pattern is relaxed or generalized by allowing searching window and even allowing backward lookup.

**Definition 2.** Let ***P***_(_*_δ_^g^*_,_*_δ_^s^*_)_ be a **specified sequential pattern set**, a set of the sequential patterns satisfying the length (***δ^g^***)– and the support (***δ^s^***)–specific condition,

***P***_(_*_δ_^g^*_,_*_δ_^s^*_)_ = { ***p*** | |***p***_g_| = *δ^g^*, |***p***_s_| ≥ *δ^s^* }.

**Figure 5:**
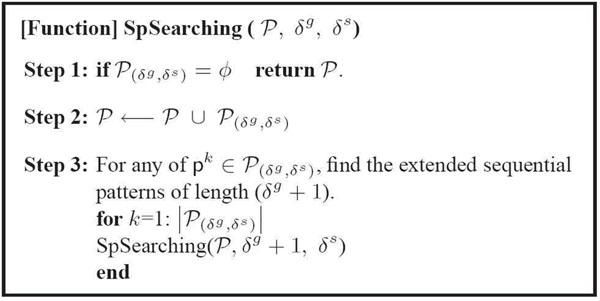
**Sequential Pattern Mining Algorithm**. Sequential Pattern Mining Algorithm

Based on the definitions above, Fig.5 presents the algorithm to generate the sequential pattern set ***P***. The algorithm, SpSearching, performs the depth-first-search (DFS) using recursion, which explores a longer pattern first. Initial calling SpSearching with ***P*** = ***P***_(1,_***_u_***_)_, ***δ^g^*** = 1, and ***δ^s^*** = ***u***, returns the set ***P***. By eliminating the patterns having shorter length than the minimum length ***l***, ***P*** = ***P*** \ { ***p*** ∈ ***P*** | |***p***_g_| <***l*** }, we obtain the final set ***P***. For further review, refer to [15,20].

The sequential patterns found by Definition 1 can vary with the parameters related to searching window, *w_b_* and *w_f_* . We show the following three special cases and present an example accordingly.

**Case 1 (searching with no window)*** g^k^* is added at the end of the reference *g^i^* if *k* satisfies *k* ∈ ( *i*, *n* ]. It results from setting *w_b_* = 0 and *w_f_* = *n−i*.

**Case 2 (searching only by forward lookup)*** g^k^* is added at the end of the reference *g^i^* if *k* satisfies *k* ∈ ( *i*, *i* + *w_f_* ] where *w_b_* = 0 and 1 ≤ *w_f_* ≤ *n − i*.

**Case 3 (searching by backward- and forward lookup)*** g^k^* is added at the end of the reference *g^i^* if *k* satisfies *k* ∈ [ *i−w_b_*, *i* + *w_f_* ], *k* ≠ *i* where 0 ≤ *w_b_* ≤ *i − 1* and 1 ≤ *w_f_* ≤ *n − i*.

**Figure 6:**
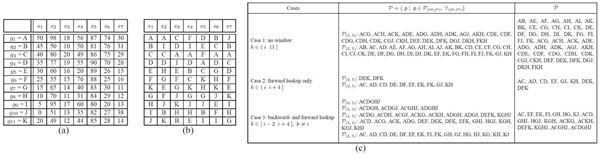
**Example of sequential patterns.** Example: (a) an expression matrix, ***E*** ∈ ℜ^|^***^G^***^|×|^***^S^***^|^ where |***G***| = 11 and |***S***| = 7, (b) a sequence database, ***D***, and (c) the set of sequential patterns, ***P***, identified by each of the three cases. The search parameters are set to ***u*** = 5, ***l*** = 2, ***w_f_*** = 4, and ***w_b_*** = 2.  is the summary set eliminating trivial patterns which are enclosed by other patterns. Compared with Case 1, Case 3 searches long sequential patterns. All the patterns found from Case 1 have the length of 2 or 3 (|***p_g_***| = 2 or |***p_g_***| = 3), whereas 27 out of the 43 patterns found from Case 3 have longer length than 3 (|***p_g_***| ≥ 3). Note that the longer patterns are more likely to have biological implication than the shorter ones which can be found by chance. Compared with Case 2, Case 3 shows the effect of backward lookup. By allowing trivial switch between consecutive elements in a sequence, one can still identify sequential patterns despite innate noise in data, e.g., experimental noises in a microarray matrix.

We can see that Case 3 rephrases Definition 1 including the others as the special cases of it. And all of the cases allow the gap between *g^i^* and *g^k^* in forward direction. Case 1 is the most strict definition for sequential pattern. In Case 2 or 3, one can set the size of searching window to be less than the full length of the sequence, which consequently leads to a more relaxed definition than Case 1. In general applications of sequential pattern mining, the number of attributes (corresponding to |***G***| in our notation) is relatively trivial to the number of samples (e.g., ***|S|* >>*|G|***). Therefore, it is not so seriously taken as infeasible to explore the |***G***|–sized sequences without restricting the searching scope. However, in the problem of finding gene sequential patterns from an expression matrix, one faces with the opposite situation (e.g., ***|S|* <<*|G|***). To the best of our knowledge so far, it easily becomes an intractable problem. Finally, Case 3 allows backward lookup when compared to the others. This relaxation particularly benefits when the sequence is not strictly ordered. It is even more preferable for microarray gene expression data since the orders of genes can often be switched by trivial difference in expression value, but the difference might be incurred by inherent or experimental noise. By allowing *g^k^* to precede *g^i^*, one can find more robust sequential patterns. Fig.6 exemplifies the difference of the three special cases.

### Definition of regulatory module

A regulatory module (RM), co-regulated genes and their co-regulating samples, can be identified from the sequential pattern ***p*** ∈ ***P*** found by SpSearching (Fig. 5).

**Definition 3.** A regulatory module, *m* is identified from a sequential pattern ***p*** as:

***m***= ( ***m^G^*** , ***m^S^*** )

where ***m^G^*** = {***g*** | ***g*** ∈ ***p_g_***} and ***m^S^*** = {***s*** | ***s*** ∈ ***p_s_***}.

The ***m^G^*** contains co-regulated genes, and therefore the functional theme in ***m*** can be achieved by annotation enrichment for the ***m^G^***. On the other hand, the ***m^S^*** contains the corresponding samples to the genes, and hence the conditional theme of the co-regulate genes in ***m***. Note that the definition of RM allows non-exclusive membership (i.e., a sample or a gene can belong to several sequential patterns).

### Types of inter-module relations

Given different RMs, we can infer the inter-module relation. The relation of two modules can be measured by the degree of overlap between the genes (or between the samples). Given ***m*** and ***m′***, let ***X_g_*** and ***X_s_*** denote the degrees of overlap between the genes and between the samples, respectively.

Where ***m***= ( ***m^G^*** , ***m^S^*** ) and ***m′*** = ( ***m^G'^***, ***m^S'^*** ). Based on the two dimensional degrees of overlap between ***X_g_*** and ***X_s_***, we can characterize the inter-module relation into the following four types; two modules are:

**Type 1: independent** when both ***X_g_*** and ***X_s_*** are low,

**Type 2: conditionally co–regulated** when ***X_g_*** is high but ***X_s_*** is low,

**Type 3: separately co–regulated** when ***X_g_*** is low but ***X_s_*** is high, and

**Type 4: similar** when both ***X_g_*** and ***X_s_*** are high.

## Authors' contributions

MK and HS implemented the method, analyzed the data and wrote the manuscript. TSC analyzed the data. JGJ improved the manuscript writing. JHK supervised the study.

## Competing interests

The authors declare that they have no competing interests.

## Supplementary Material

Additional file 1 **Details of benchmark** Details of benchmark, such as the generation of simulation data and the parameter selection Click here for file

Additional file 2 **GO-slim terms and SMD-sample categories** Gene Ontology (GO) slim terms (of Biological Process) defined by SGD and SMD categories which describe the experimental context of microarray samples Click here for file
