# Supplementary material for: Extracting regulatory modules from gene expression data by sequential pattern mining
Source: BMC Genomics. 2011 Nov 30;12(Suppl 3):S5. doi: 10.1186/1471-2164-12-S3-S5 (PMC3333188; doi:10.1186/1471-2164-12-S3-S5)

**1. Generation of simulation data**

Five base matrixes generated by random sampling from *N*(0, 10) – a normal distribution with mean 0 and standard deviation 10 – with a size of 50 rows and 50 columns. For each matrix, five patterns with a size of 5 rows and 5 columns are embedded in such a way that selected cells are increasingly ordered in row-wise. For noise simulation, five levels of Gaussian noises — mean 0 and standard deviation 0.1, 0.2, 0.3, 0.4 and 0.5 – are added to the matrixes. In following heatmaps for one of five matrixes, we can see order-preserving patterns persist throughout all experimental noise levels.

**2. Algorithm implementation**

We implement a sequential pattern mining algorithm. The source code is available as Python code at the supplementary web page. For OPSM, we used an implementation in BicAT (<http://www.tik.ee.ethz.ch/sop/bicat/>) with default parameters.

**3. The effect of forward window (Wf)**


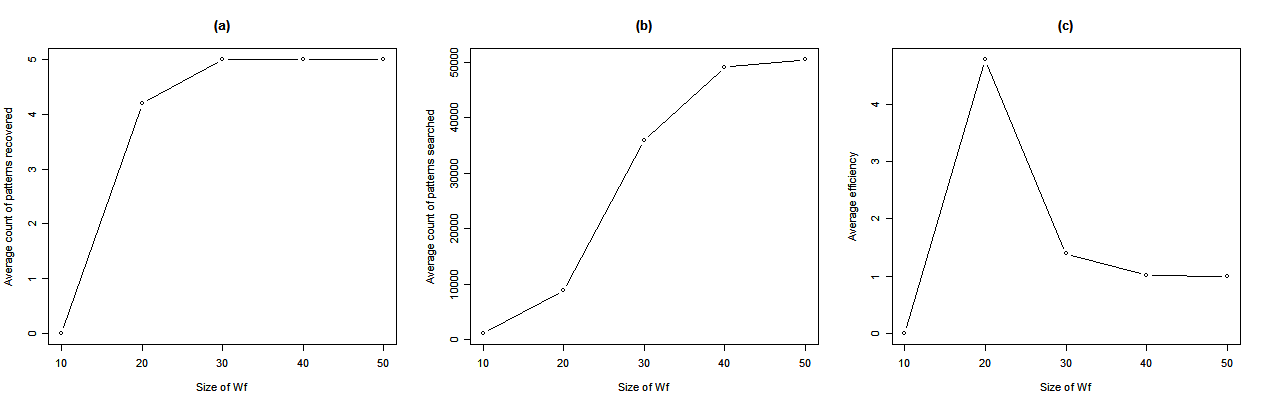


We applied the SPM algorithm with varying Wf to the simulation data with zero noise. Other parameters are set as window backward=0, minimum supports=10, minimum length=10. As Wf gets smaller, the number of patterns searched declines much faster (b) then the number of patterns recovered (a). Thus we have the best performance with Wf=20 (c).

**4. The effect of Wb and noise**


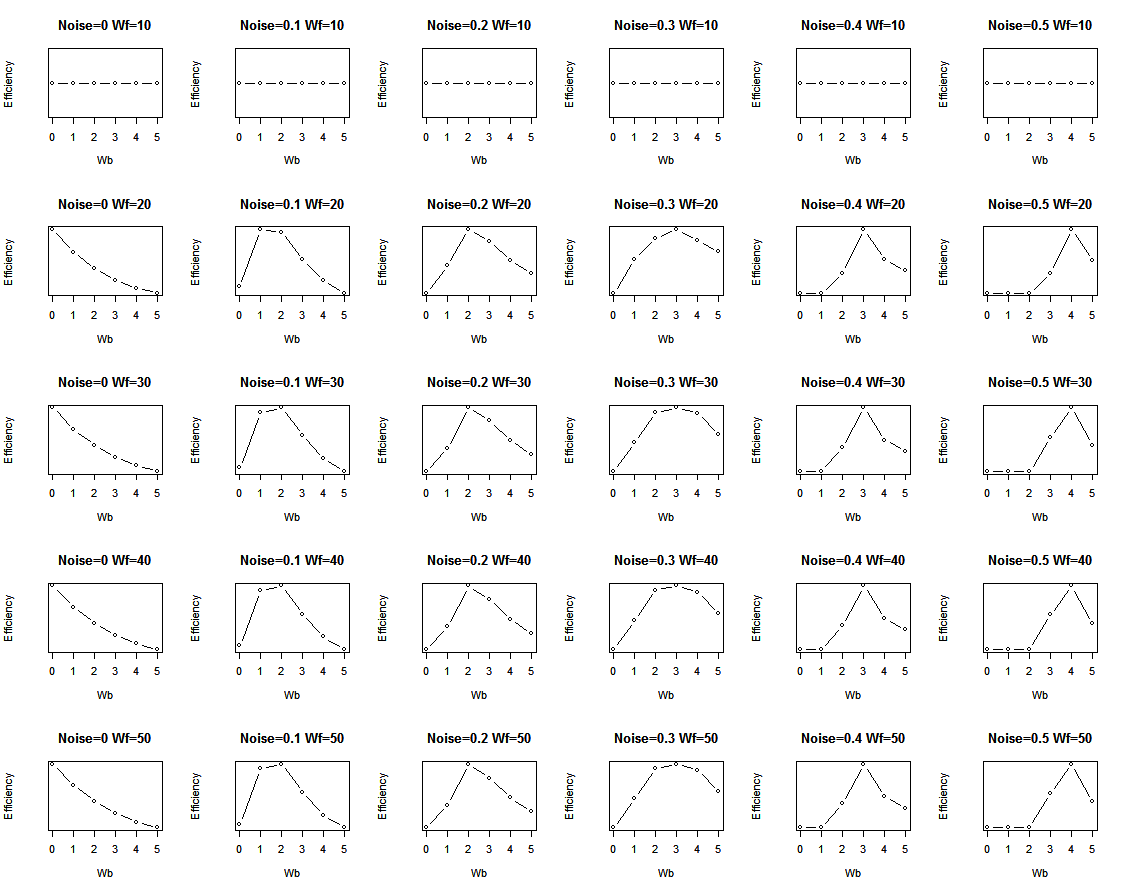


For fixed Wf and noise, we evaluate the effect of Wb. As can be seen in the first column, for data without noise, Wb only decreases efficiency by increasing the number of patterns searched. But as larger noise exists in data we need larger Wb for efficient discovery of patterns.

**5. Sensitivity benchmark (Fig.3a ) in other parameters settings**

SPM-naïve – circle, SPM-window – triangle, and OPSM – cross.


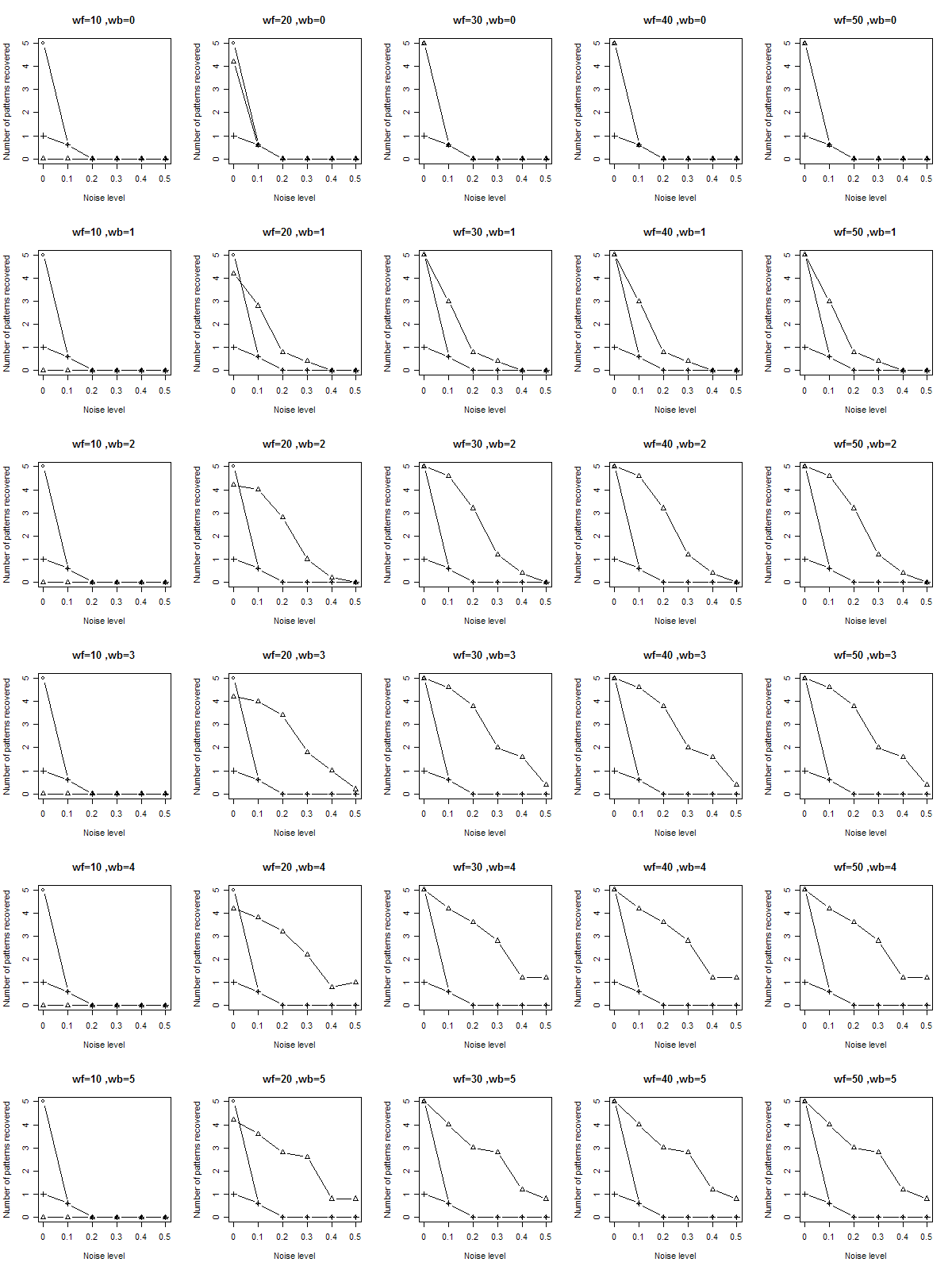


**6. Complexity benchmark (Fig. 3b) in other parameter settings**

SPM-naïve – circle and SPM-window – triangle.


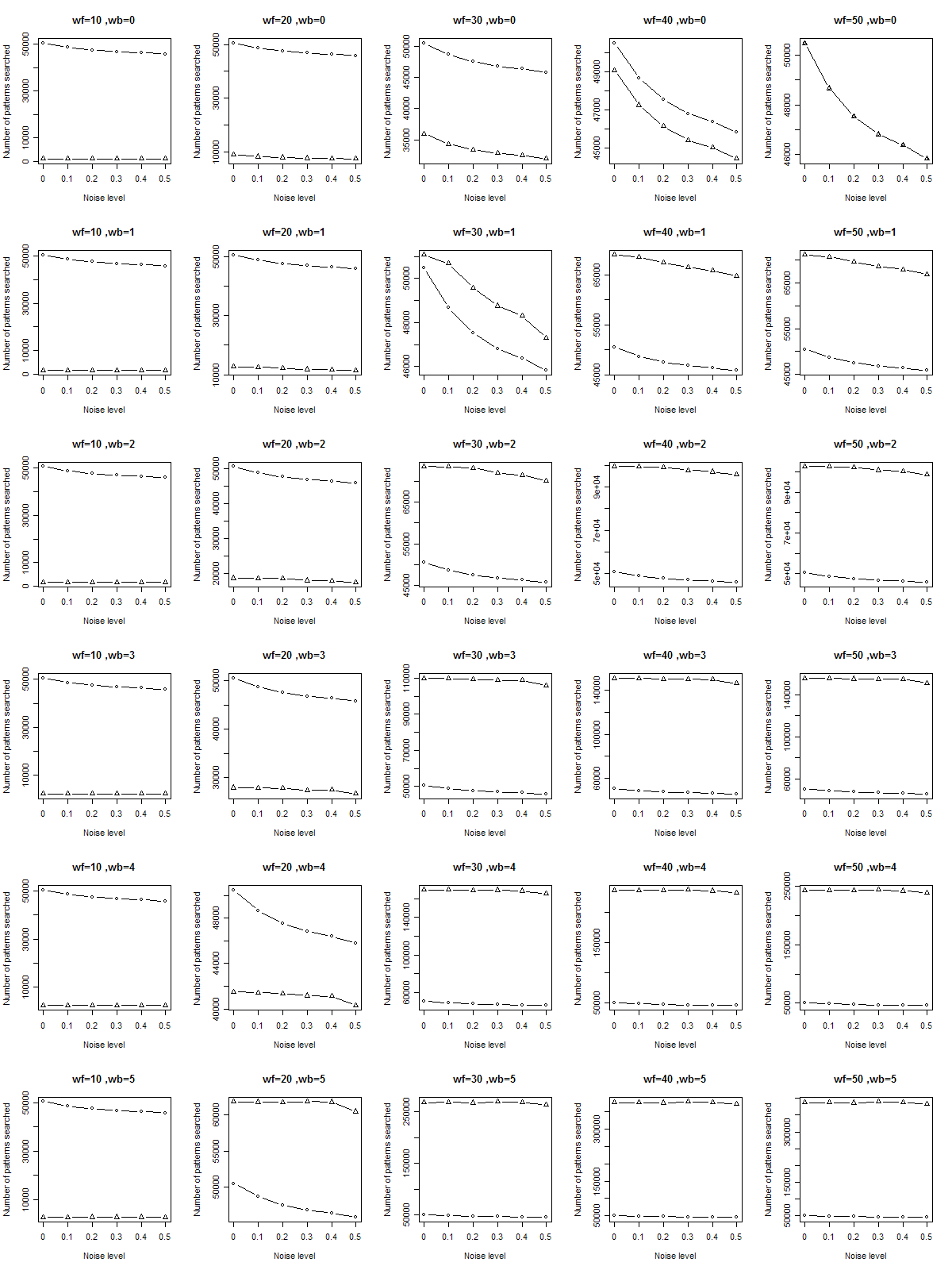


**6. Efficiency benchmark (Fig. 3c) in other parameter settings**

SPM-naïve – circle and SPM-window – triangle.


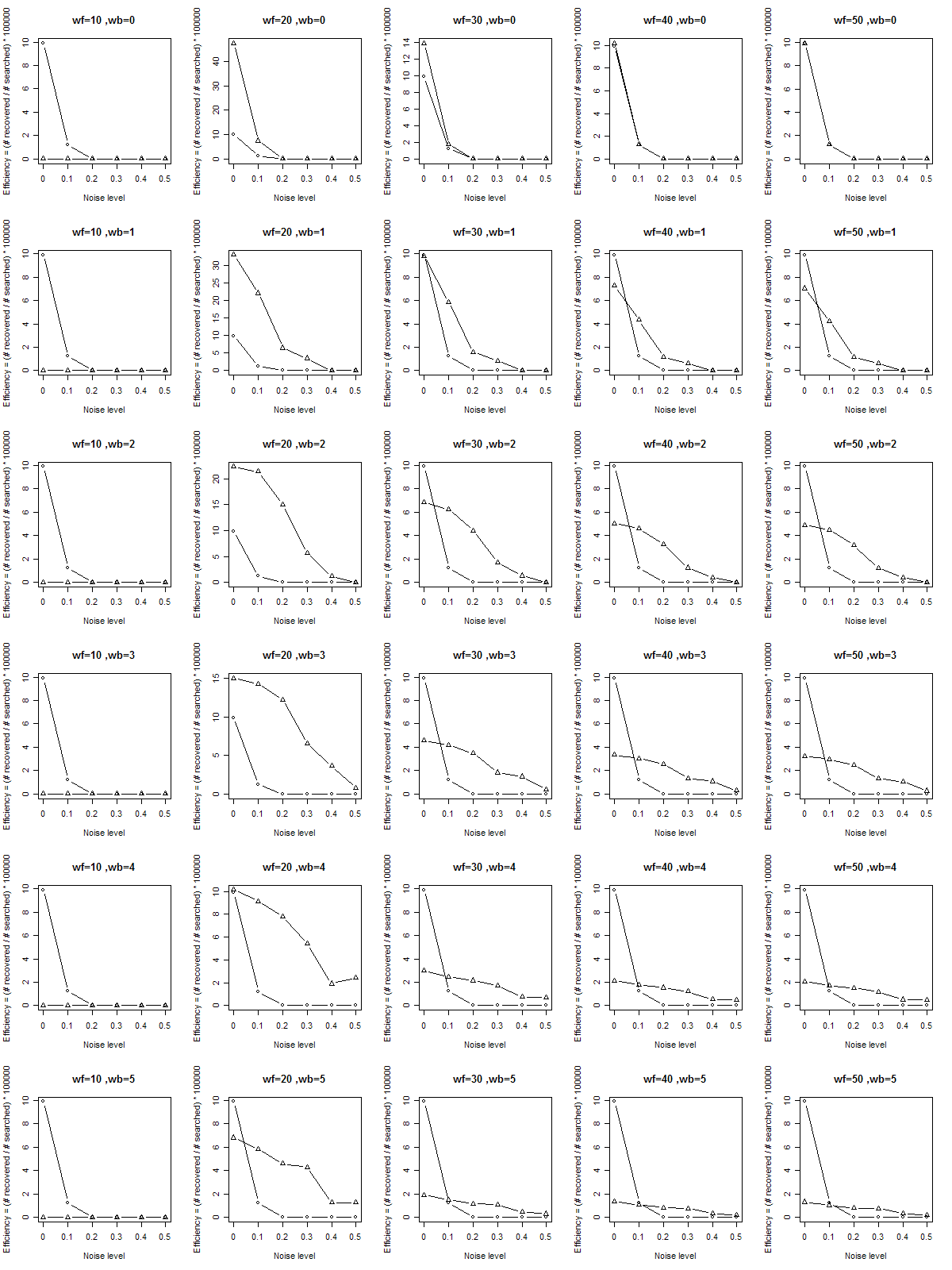

Supplement: Additional file 1 — Details of benchmark Details of benchmark, such as the generation of simulation data and the parameter selection [file 1471-2164-12-S3-S5-S1.doc]
